# Superscan Pattern on Bone Scintigraphy: A Comprehensive Review

**DOI:** 10.3390/diagnostics14192229

**Published:** 2024-10-06

**Authors:** Emran Askari, Sara Shakeri, Hessamoddin Roustaei, Maryam Fotouhi, Ramin Sadeghi, Sara Harsini, Reza Vali

**Affiliations:** 1 Nuclear Medicine Research Center, Mashhad University of Medical Sciences, Mashhad 13944-91388, Iran; emran.a69@gmail.com (E.A.); sarash886@gmail.com (S.S.); roustaeihessam@gmail.com (H.R.); sadeghir@mums.ac.ir (R.S.); 2 Division of Molecular Imaging and Theranostics, Department of Nuclear Medicine, University Hospital, Paracelsus Medical University, 5020 Salzburg, Austria; 3 Advanced Diagnostic and Interventional Radiology Research Center (ADIR), Cancer Institute, Imam Khomeini Hospital Complex, Tehran University of Medical Sciences, Tehran 1419733141, Iran; maryamfotuhi.m@gmail.com; 4 BC Cancer Research Institute, Vancouver, BC V5Z 1LL3, Canada; sharsini@bccrc.ca; 5 Department of Nuclear Medicine, The Hospital for Sick Children, University of Toronto, Toronto, ON M4N 3M5, Canada

**Keywords:** superscan, bone scintigraphy, metastasis, metabolic bone disease, prostate cancer

## Abstract

Background/Objectives: The superscan pattern is a characteristic finding on bone scintigraphy, associated with a variety of metabolic bone diseases, malignancies, and other conditions. This pattern is characterized by a diffuse and intense uptake of radiotracer throughout the entire skeleton. Despite being a relatively rare finding, the superscan pattern can have significant clinical implications. Methods: This comprehensive review summarizes the available literature on the superscan pattern, focusing on its pathophysiology, clinical significance, and differential diagnoses. Relevant studies and case reports were analyzed to outline the diagnostic challenges associated with the interpretation of bone scintigraphy featuring the superscan pattern. Results: The literature highlights the clinical significance of the superscan pattern in various metabolic and oncologic conditions. Misinterpretation of this pattern can lead to diagnostic challenges, especially in distinguishing it from other pathologic conditions. Differential diagnosis remains crucial in the accurate interpretation and subsequent management of patients with this finding. Conclusions: This review provides a comprehensive overview of the superscan pattern on bone scintigraphy, aiming to assist clinicians in recognizing and managing this rare yet clinically important finding.

## 1. Introduction

Bone scintigraphy is a sensitive imaging modality (80–100%) for detecting osteoblastic bone metastases, which can present as solitary or multiple foci, or as diffuse increased tracer uptake throughout the skeleton. The “super bone scan”, “superscan”, or “beautiful bone scan” is a distinct pattern of skeletal radioisotope uptake potentially mimicking a normal scan in the eyes of an inexperienced reader [1,2,3]. This pattern is caused by diffuse active bone formation and can occur in the setting of either diffuse bone metastases or metabolic bone diseases [3].

The differential diagnoses for a superscan pattern on skeletal scintigraphy include diffuse osteoblastic metastases commonly seen in patients with prostate or breast cancer, as well as various metabolic bone diseases, such as renal osteodystrophy, osteomalacia, severe primary hyperparathyroidism, and hyperthyroidism. A prolonged delay before imaging can also mimic a superscan appearance. It is essential to accurately recognize the pattern of uptake and differentiate between superscans caused by metabolic abnormalities and those originating from osteoblastic malignancies [1]. The term superscan is also applied to other radiotracers, such as 2-deoxy-2-[^18^F]fluoro-D-glucose (^18^F-FDG), which is associated with distinct etiologies [2].

Some nuances exist that could cause a superscan to be mistaken for a normal bone scan, potentially impeding the diagnosis and treatment of cancer patients [4]. Therefore, physicians must be aware of superscan patterns and their subtypes to accurately diagnose and manage cancer patients. This study aims to comprehensively review different aspects of superscan patterns to provide physicians with a better understanding of this entity. We also review all teaching cases with a superscan pattern in our local centers (n = 3) in the past decade.

## 2. Definition

**Figure 1:**
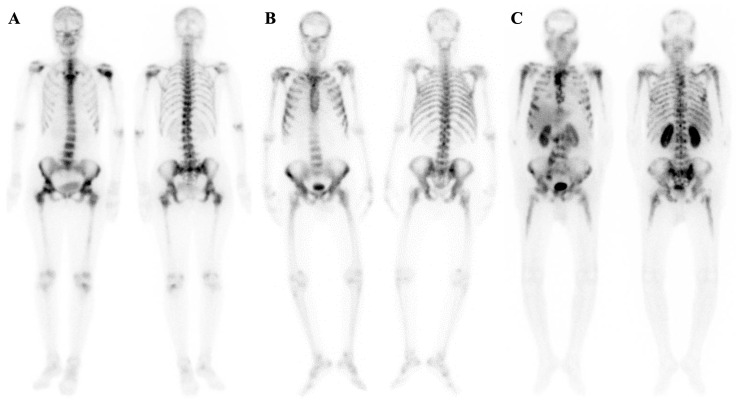
Typical cases of superscan pattern are shown in patients with metastatic breast cancer (**A**) and metastatic castration-resistant prostate cancer (**B**). The latter was confirmed through whole-body ^99m^Tc-HYNIC-PSMA SPECT/CT imaging (**C**). In some cases, bone scintigraphy, PSMA imaging, and post-treatment scans using ^177^Lu-PSMA-617 show nearly identical visual appearances.

A superscan is characterized by diffuse, intense, and relatively symmetrical osseous radiotracer uptake, often with faint or absent tracer uptake in the urinary system and soft tissues. Renal radiotracer uptake in a superscan is lower than that in adjacent ribs, and bone metastases may not appear as distinct foci of metabolically active lesions [1] (Figure 1).

According to certain studies, visualizing a minimum of three distinct sacral foramina can serve as an ancillary finding to support the diagnosis of a superscan [5]. Other studies define the involvement of more than 75% of ribs, vertebrae, and pelvic bones as a superscan pattern equivalent. This classification, known as the extension of disease (EOD), was initially described by Soloway et al. and later widely adopted in many prostate cancer research studies. EOD type-4 is equivalent to the superscan pattern [6,7,8].

## 3. History and Prevalence

The superscan pattern was first described by Osmond et al. in 1975 [9]. In the same year, Sy et al. referred to it as “absent or faint kidney” [10], which may have been influenced by earlier case reports of false-negative bone scans with extensive bone involvement [11,12].

The prevalence rate of the superscan varies from 0.2% to 0.81% of all bone scans and up to 1.3% in oncological patients [1,13,14]. Among patients with prostate cancer and bone metastasis, the reported prevalence varies widely, ranging from 4% to 17% [1,15,16].

## 4. Diagnostic Challenges

As previously stated, misinterpretations can happen [3,4,15], as the scan quality is often considered “too good”, and misleadingly reassuring, in these patients [17]. Moreover, in patients with superscan patterns, fractures or lytic areas may remain undetected [18]. Additionally, the superscan pattern can lead to unexpectedly high and abnormal increases in bone mineral density [19]. The bone scan index may also be underestimated in such patients [20].

In general, in approximately 6% of bone scans, the kidneys are either not visible or exhibit faint tracer uptake. However, it is worth noting that more than 90% of these cases are attributed to underlying renal disease rather than the superscan [21]. Although background activity is high in these cases, it can potentially masquerade as a superscan pattern if the patient undergoes renal dialysis between the radiotracer injection and imaging, leading to a false diagnosis [22]. On the other hand, in children, the lack of uptake in the kidneys could be due to intense epiphyseal uptake or cellulitis. In patients with normal renal function, the ratio of radiotracer retention in the bone to kidney is 2:3. In cases of superscan, this ratio is significantly altered to up to 6:1, and urinary excretion of the radiotracer is markedly diminished [4].

Quantitative assessments based on the “T12 index” have been shown to be more accurate in recognizing the superscan pattern compared to visual assessment alone. One of the pioneering quantitative studies on superscan, published by Constable et al. in 1993, employed the T12 index to define the superscan and revealed significant differences between patients with non-metastatic or oligometastatic prostate cancer and those exhibiting the superscan pattern. Specifically, the T12 index count ratio of the T12 vertebra to soft tissue on posterior views was significantly different in these patient groups with a range of 2.4–7.8 in patients with non-metastatic or oligometastatic prostate cancer and 17–24 in those with the superscan pattern. These images and calculations were obtained using a standard time interval of 3–5 h post-radiotracer injection [15]. However, delayed imaging may lead to an increased signal-to-noise ratio and mimic the superscan pattern [4]. Unfortunately, a notable limitation of the T12 index is its poor performance in distinguishing certain metabolic disorders, such as primary hyperparathyroidism and osteomalacia, from a normal bone scan, as these disorders can also cause increased radiotracer uptake in the spine and superscan pattern, but a false-negative superscan pattern according to this index. Calculating the 24 h radiotracer retention may provide a more accurate assessment in these cases [23].

## 5. False-Negative Interpretations

**Figure 2:**
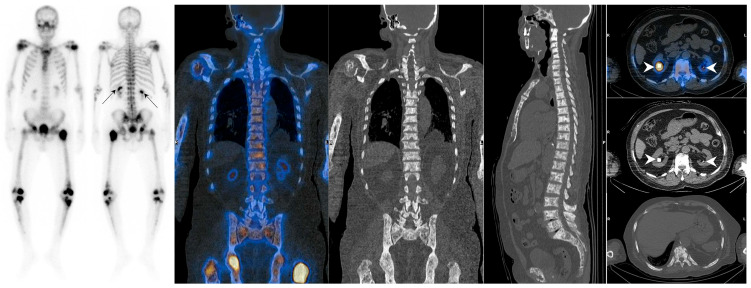
Atypical case of metastatic superscan in an 83-year-old man with metastatic prostate cancer being referred for evaluation of his response to treatment. He had been experiencing gradual pain in his lower extremities over the past 3 months, despite a decline in his PSA levels from 15 to 6.2 ng/mL. A bone scan showed increased activity in the proximal humeri, proximal and distal femora, and proximal tibiae, with activity smearing to the mid-shaft of the femur and tibia on both sides. Given his history of de novo high-volume metastatic disease and generalized bone pain, an SPECT/CT correlation was acquired. The scan revealed diffuse marrow involvement throughout the axial and appendicular skeleton (superscan equivalent). Interestingly, bilateral renal uptake (arrows) was primarily located at the site of nephrolithiasis, indicating obstructive uropathy (arrowheads).

In which scenarios should a superscan be considered despite kidney visualization? Any factor that leads to abnormally increased ^99m^Tc-MDP uptake by the kidneys can potentially limit the detection of a superscan pattern (Figure 2). The term “hot kidneys” in a bone scan is used when kidney uptake exceeds that of the adjacent vertebra. Kidneys with high uptake are seen in 0.5–2% of bone scans [24]. The differential diagnoses for hot kidneys include obstructive uropathy (especially in patients with prostate cancer) [3,15], hypercalcemia, hyperparathyroidism, chemotherapy [3,25], iron overload (as in sickle cell anemia), acute kidney injury (due to acute tubular necrosis (ATN) and non-steroidal anti-inflammatory drugs), recent radiotherapy, use of anticonvulsant drugs, and aluminum breakthrough [26].

**Figure 3:**
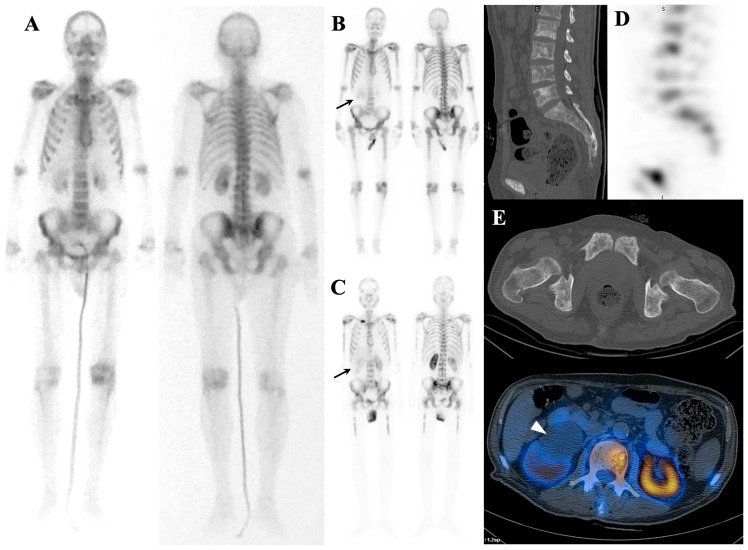
Atypical case of metastatic superscan in a 67-year-old man with biochemical persistence following radical prostatectomy (Gleason score 4 + 5 and initial PSA = 64 ng/mL). The patient was referred for ^99m^Tc-HYNIC-PSMA SPECT/CT and repeat bone scan due to highly elevated PSA levels, 3 months post-prostatectomy (PSA = 42 ng/mL). The initial bone scan was reported as negative (**A**), but a retrospective review revealed subtle, suspicious foci in the left lowest rib and right sacroiliac region. Subsequently, the recent bone scan (**B**) suggested a superscan, although the visualization of the kidneys was uncertain (black arrow). A few days later, PSMA imaging (**C**) was performed, unveiling widespread skeletal metastases (**D**) and indicating a poorly functioning right kidney and obstructive uropathy (black arrows in (**B**,**C**); white arrowhead in (**E**)). Notably, the right kidney showed more prominent abnormalities. It is important to note that non-visualization of the kidneys is not a prerequisite for a superscan and should be considered in prostate cancer patients with a long-standing history of benign prostatic hyperplasia, where kidney uptake may be preserved despite extensive bone metastases. This specific type of superscan is sometimes referred to as a sub-superscan or forme-frustré of a superscan.

Some tumors may infiltrate the bone marrow without eliciting a remarkable generalized osteoblastic reaction. The reason for this phenomenon is not necessarily attributed to a milder disease; instead, intratrabecular metastases are often unable to trigger a substantial osteoblastic response. Therefore, the typical appearance of a superscan may not be evident in these patients [27]. This particular mode is referred to as a forme-frustré superscan or sub-superscan [3] (Figure 3). Reconstructing hybrid single-photon emission computed tomography/computed tomography (SPECT/CT) images using the xSPECT bone algorithm (20 iterations; one subset; and 20 mm Gaussian filter) may offer improved diagnostic capabilities for challenging cases compared to the standard Ordered-Subset Expectation–Maximization (OSEM) algorithm (eight iterations; four subsets; and 12 mm Gaussian filter) [28]. Attention to focal uptake in the ribs or distal long bones may be helpful, although further research is needed to definitively establish the clinical utility of these findings in supporting a superscan diagnosis [29].

**Figure 4:**
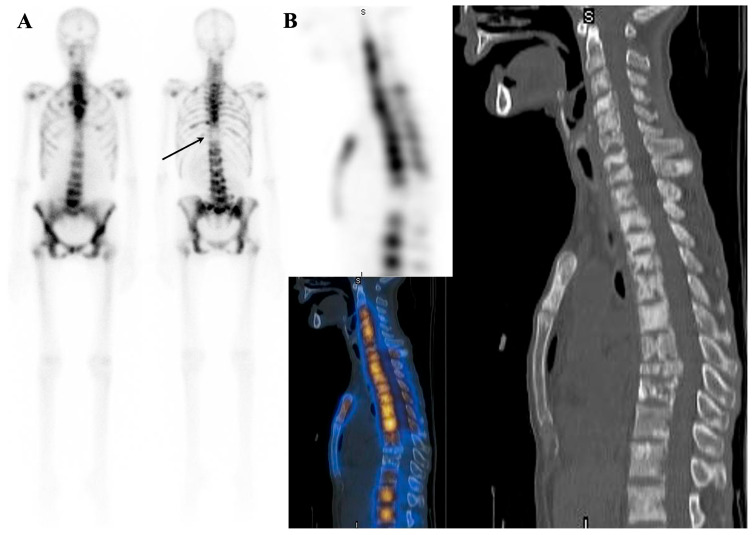
Atypical case of metastatic superscan in a 52-year-old woman with a history of breast cancer, prior radiation therapy, and recent paraplegia. In the recent bone scan, the patient exhibits multiple non-homogeneous foci of increased tracer uptake throughout the axial skeleton, accompanied by the absence of kidney visualization—a pattern consistent with a metastatic superscan (**A**). Additionally, a photopenic area is observed in the lower thoracic spine (arrow). Fused SPCET and CT images provide further insights, revealing a compression fracture in the T9 and T10 vertebrae, which coincides with the region previously treated with radiotherapy (**B**). This compression fracture exerts a compressive effect on the spinal cord.

Rare cases of slow-growing tumors with osteoblastic reaction and extensive bone marrow involvement may not exhibit a superscan appearance [30]. It is also possible that the patient’s superscan view may contain areas of decreased uptake on the bone scan, which could be attributed to prior radiotherapy, concomitant lytic lesion, or bone infarction [31] (Figure 4).

## 6. Elucidating the Mechanisms Underlying a Superscan Pattern on Bone Scintigraphy

The main reason for the uptake of the bone-seeking radiopharmaceuticals in the bones is thought to be chemisorption. However, the uptake can be altered in cases of increased bone blood flow [32], amplified osteoid formation [33], an expanded mineralization surface [34], accelerated bone turnover [35], and the presence of immature collagens [36]. Pathologic conditions can lead to a superscan appearance by any of the mechanisms mentioned above. Additionally, severe bone marrow involvement in metastatic patients can also contribute to the development of such scan patterns [37]. Decreased renal uptake could be due to underexcretion of urinary phosphate secondary to increased bone uptake, renal failure, or both [10]. Elevated serum phosphate levels can lead to enhanced hydroxyapatite formation and hence increased uptake of these agents [38]. Table 1 summarizes the underlying mechanisms.

Superscan patterns have been reported with various radiotracers, including ^18^F-FDG [2], for which the steal phenomenon may provide a plausible explanation. In the context of ^18^F-FDG PET, the superscan pattern is characterized by reduced pronounced reduction in physiological uptakes in the brain, heart, and kidneys. The superscan pattern seen in ^18^F-FDG PET can be encountered in several differential diagnoses, including chemotherapy and marrow rebound [2], recent colony-stimulating factor injection [2], fever and infection (due to secretion of interleukins) [2], primary and secondary hyperparathyroidism [39], neuroendocrine tumors [40], lung cancer [41], prostate cancer [42], renal cell carcinoma [43], leukemia [44], lymphoma [45], multiple myeloma [46], hemophagocytic lymphohistiocytosis [47], rhabdomyosarcoma [48], and neuroectodermal tumors [49].

**Figure 5:**
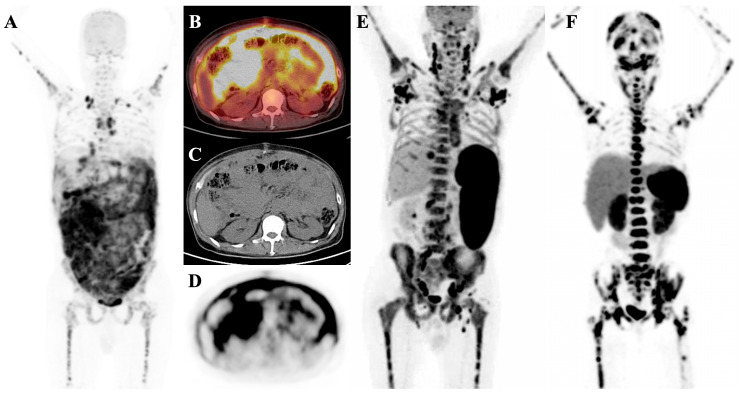
(**A**–**D** and **E**) Two representative cases of high-grade lymphoma are presented with extensive involvement leading to suppressed cardiac and brain metabolic activity (FDG superscan). In the first case (**A**–**D**), diffuse bone marrow involvement is noted along with peritoneal lymphomatosis. The second case (**E**) illustrates diffuse bone marrow, splenic, and nodal involvement. (**F**) A case with refractory neuroblastoma with diffuse marrow involvement having intense somatostatin receptor avidity. The patient received two doses of ^177^Lu-DOTATATE, given his history of mIBG-negative disease and tumoral recurrence following bone marrow transplant. However, disease progression was inevitable.

Furthermore, superscan patterns have been observed in meta-iodobenzylguanidine (MIBG) and somatostatin receptor (SSTR) imaging, including cases of pheochromocytoma [50], paraganglioma [51], and neuroblastoma [50,52] with MIBG and pheochromocytoma [50], neuroblastoma [53], neuroendocrine carcinoma [54], and breast cancer with SSTR [55]. In addition to these, superscan patterns have been identified in imaging with other radiotracers, such as ^18^F-choline PET in patients with lymphoproliferative disorders [56], metastatic prostate cancer, and rebound after chemotherapy [57]; ^18^F-fluciclovine PET [58] and prostate-specific membrane antigen (PSMA) SPECT/PET in patients with prostate cancer [59]; carbon-11 (^11^C)-methionine PET in polycythemia vera [60]; ^18^F-sodium fluoride (NaF) PET in renal osteodystrophy [61]; gallium-68 (^68^Ga)-Pentixafor PET in multiple myeloma [62]; and ^68^Ga-fibroblast activation protein inhibitor (FAPI) PET in myelofibrosis and breast cancer [63,64] (Figure 5). Additionally, the superscan pattern has also been reported in diffusion-weighted magnetic resonance imaging (MRI) in patients with lymphoma [65].

## 7. Etiologies

**Figure 6:**
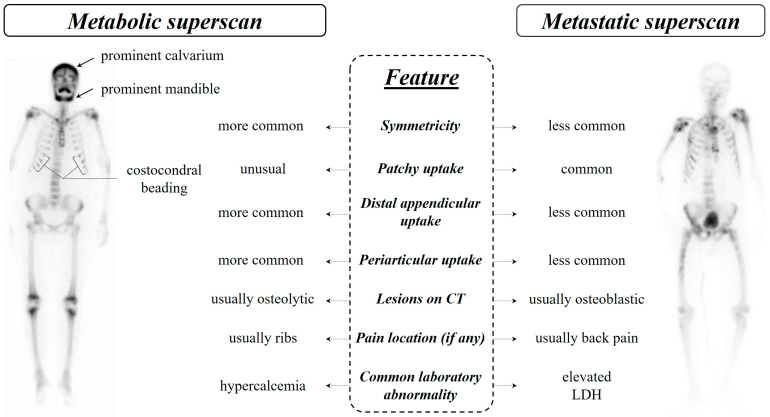
Comparison of metabolic and metastatic superscan (for more details, see also Section 7.3).

Superscan etiologies can be categorized into metabolic, hematologic, and metastatic entities, as summarized in Table 2. When approaching a patient with a prior history of cancer, it is important to first consider the metastatic causes [66]. Additionally, in patients with low-risk cancer and no evidence of recurrent disease, a metastatic superscan pattern may warrant suspicion of a double carcinoma [67] (Figure 6).

### 7.1. Metastatic Superscan

Metastatic superscan patterns are usually representative of diffuse carcinomatosis in the bone marrow and are accompanied by anemia, lumbar pain, bleeding diathesis, disseminated intravascular coagulation, and poor prognosis [99]. According to Paget’s seed and soil theory, certain cancer cells have a preference for settling in certain sites [100]. Prostate, breast, and lung carcinomas are known to be bone marrow (BM)-tropic [101]. Prostate cancer is the most common cause of metastatic superscan [15]. Apart from BM-tropism [102], other mechanisms have been postulated for such observations in patients with prostate carcinoma. As an example, prostate cancer cells can induce increased uptake of bone-seeking radiopharmaceuticals by secreting osteoblast-stimulating factors and stimulating collagen production [103]. These patients are often elderly and may suffer from some degree of renal impairment, leading to hypocalcemia secondary to osteoblastic metastases and diminished renal absorption of bone-seeking radiopharmaceuticals [104]. Considering these changes, these patients are prone to secondary hyperparathyroidism [105]. The combination of these factors contributes to the increased prevalence of superscan among patients with prostate cancer, sometimes presenting a headless appearance, which can be justified by the low tendency of prostate metastasis to the skull [106].

Other common causes of metastatic superscan include breast cancer (4.5–4.7% of cases) [73,107], lung cancer [1], gastric cancer (2.6% of the cases) [77], neuroblastoma [87], nasopharyngeal cancer (1.5% of the cases) [84], urinary tract and bladder carcinoma [68], medullary thyroid cancer [1], colorectal cancer [66], Ewing sarcoma [1], esophageal cancer [1,66], salivary gland tumors [1], melanoma [94], pheochromocytoma [50], metastatic medulloblastoma [98], and intracranial glioma [96,97].

Bone metastasis has been reported in 2–17.5% of gastric cancer cases, with the metastasis often being osteolytic [108]. Additionally, there are reports of a variant of gastric adenocarcinoma (referred to as Borrmann type 4) that produces alkaline phosphatase and is associated with osteoblastic lesions and a superscan appearance. The overall prevalence of this variant is unknown [99,109].

Moreover, nasopharyngeal cancer cases with superscan are often associated with elevated levels of immunoglobulin A (IgA) or IgG antiviral capsid antigen, as well as liver metastasis [110]. Additionally, in less than 2% of cases, gliomas can exhibit extracranial metastasis. There have been case reports documenting superscans resulting from bone marrow infiltration by this tumor [111].

#### 7.1.1. Superscan Pattern in Patients with Prostate Cancer

These patients with a superscan pattern on bone scintigraphy are usually aged 60-65 years old with an initial stage of III or IV (with 12.5% having visceral metastasis and 20.8% having lymphatic metastasis). High levels of prostate-specific antigen (PSA) and alkaline phosphatase are often observed in these patients [1], with one study reporting a superscan pattern in all patients with a PSA > 100 ng/mL [112], and alkaline phosphatase flare (which is associated with a poor prognosis) is more likely to occur [113]. Chronic pain is also commonly reported in patients with a superscan pattern, as they are more likely to have a higher Gleason score [1] and more rapid progression toward metastatic castration-resistant prostate cancer (mCRPC) [114]. Additionally, these patients are at a higher risk of cord compression [6] and probably have a higher prevalence of hypocalcemia [115], impaired bone marrow function, and hemoglobin drop [116]. It is noteworthy that, although hypocalcemia is associated with a higher load of bone metastasis, hypercalcemia is associated with a worse prognosis. This is not surprising when hypercalcemic patients often have lytic metastases, high levels of chromogranin A, lactate dehydrogenase, and bone pain, suggesting the presence of the neuroendocrine prostate cancer phenotype [117].

The presence of a superscan pattern on bone scintigraphy is a poor prognostic indicator in prostate cancer patients, with several trials excluding these patients due to their limited lifespan, inability to assess disease progression by conventional imaging, increased bone marrow susceptibility, and delayed recovery following treatment [118]. Superscan is considered a relative contraindication for bone palliative treatments, as hematotoxicity usually occurs within 4–6 weeks of treatment and recovers within 4–6 weeks, but recovery may be delayed in patients with superscan patterns [119]. There are conflicting results in response to bone palliative treatments in patients with a superscan pattern [120,121], and some recommend dose adjustments in these groups of patients [122]. According to the EANM guidelines, patients with a superscan who have an acceptable bone marrow reserve are candidates for bone palliative treatments [123]. The possibility of thrombocytopenia following treatment with radium-223 is higher in patients with a superscan pattern, and their prognosis is usually poor, with patients often dying before completing treatment cycles [124]. Therefore, treatment with radium-223 in these patients should be considered following a thorough risk–benefit assessment [125].

Despite discouraging results with bone palliative agents, multicenter retrospective studies have shown that the biochemical response in patients with a superscan pattern who underwent treatment with lutetium-177 (^177^Lu)-PSMA-617 is rather high, with 58% of patients experiencing more than a 50% decrease in PSA and one-fourth of patients showing major hematotoxicity within an acceptable range [126]. Therefore, both the EANM and SNMMI guidelines consider these patients to be eligible for PSMA radioligand therapy (RLT) if they met the other inclusion criteria [127,128]. Moreover, these patients are good candidates for alpha-RLT (e.g., by using actinium-225 (^225^Ac)) [129]. Lawal et al. showed that the risk of cytopenia following treatment with ^225^Ac-PSMA-617 in mCRPC patients with a superscan pattern or extensive bone involvement is negligible, with age, the number of treatment cycles, and the presence of renal disorders being predictive of hematologic complications following alpha-RLT [130]. Combined alpha and beta therapy has also successfully eliminated the leukoerythroblastic pattern and extensive bone marrow involvement in some cases [131]. This success is partly due to a high affinity for the radiotracer absorption, which can result in a steal phenomenon where the normal physiological uptake is significantly reduced [132]. This phenomenon is also referred to as the tumor sink effect. The presence of this pattern can potentially minimize adverse events associated with physiological uptake and enhance the efficacy of RLT by allowing for higher doses in the initial treatment cycle [133].

#### 7.1.2. Dynamic Changes in Prostate Cancer Patients with Superscan

Rapid disease progression, including the emergence of a superscan pattern, can occur in rare instances following local treatment [134]. However, in the majority of cases, the transition to a superscan pattern happens gradually during the follow-up of patients with metastatic cancer, with de novo superscan development being a relatively uncommon occurrence [135]. Once a patient has reached this stage, the scan findings typically do not regress [136]. Although the use of tyrosine kinase inhibitors in mCRPC patients may cause the regression of bone scan findings [137], the COMET-1 trial demonstrated that cabozantinib does not improve overall survival in mCRPC patients, despite showing improvement in bone scan findings [138].

On the other hand, the flare phenomenon, which occurs after treating patients with a superscan, can render the interpretation of findings challenging and may lead to indeterminate results [139]. Consequently, bone scans might be deemed less useful in some trials for evaluating disease progression [140].

### 7.2. Metabolic Superscan

Metabolic superscan is a characteristic pattern of bone scintigraphy that can be seen in a number of clinical conditions (Table 2). The association between superscans and poor prognosis is well established in cancer patients, whereas the relationship between superscans and prognosis in patients with benign diseases remains poorly understood [141].

**Figure 7:**
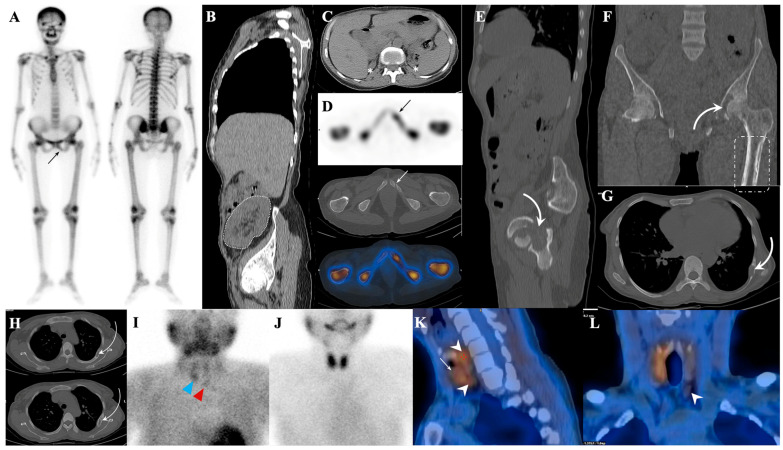
This is a typical case of metabolic superscan (**A**) in a patient with a history of renal transplantation. The transplant kidney is visible in the pelvic fossa (dashed, (**B**)) while the native kidneys appear atrophic (asterisks, (**C**)). The scan shows a focal zone of MDP uptake in the left pubic ramus, compatible with fracture (arrows, (**A**,**D**)) and multiple lytic areas (curved arrows in (**E**–**H**)) localized to the left femoral head, left acetabular roof, and multiple ribs. Additionally, a periosteal reaction is noted in the left proximal femur (dashed rectangle, (**F**)). Many of these abnormalities are not easily discernible in the whole-body bone scan. Subsequent correlative imaging using the dual tracer protocol was employed (**I**,**J**), revealing a focus of retained activity on the right side due to a thyroid nodule (blue triangle, (**I**) and arrow, (**K**)) and enlarged parathyroid glands bilaterally (red triangle, (**I**) and arrowheads in (**K**,**L**)). The final diagnosis was secondary parathyroid hyperplasia and renal osteodystrophy.

**Figure 8:**
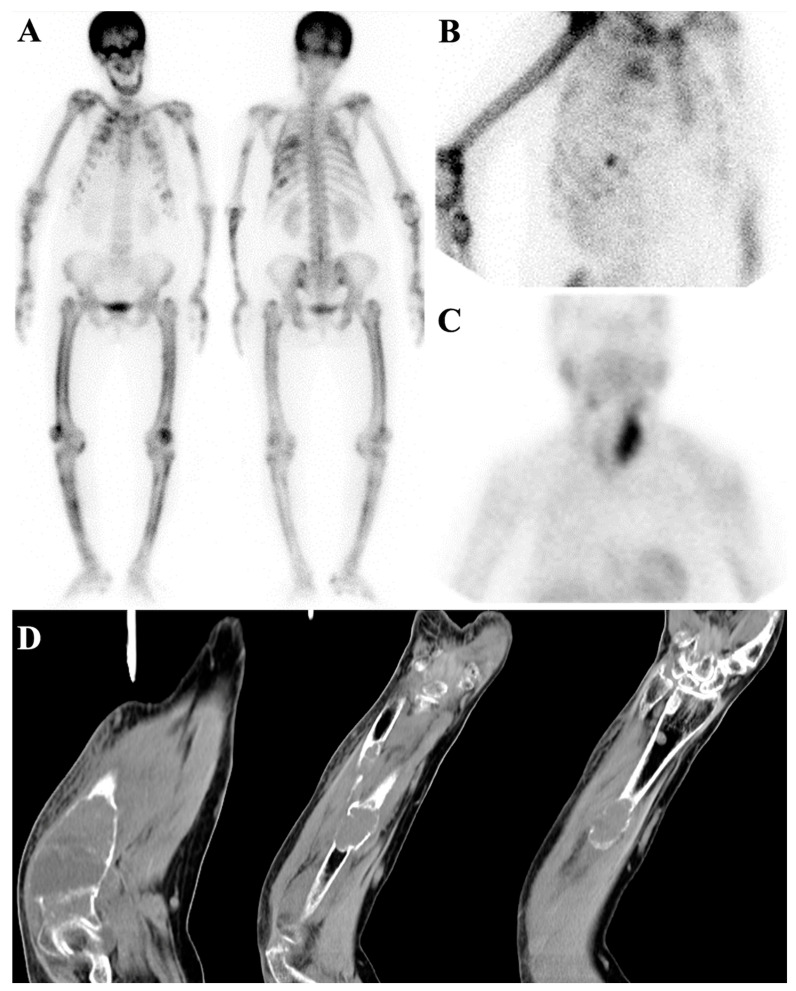
Atypical case of metabolic superscan in a patient with a history of generalized bone pain and recent swelling of the right distal humerus and proximal radius (**A**). Upon examination, the bone scan reveals bone expansion and a focal rib uptake consistent with a fracture (**B**). Parathyroid scintigraphy uncovers a sizable ^99m^Tc-sestamibi-avid lesion on the left side, suggestive of parathyroid adenoma/carcinoma, which is later confirmed to be parathyroid carcinoma (**C**). Correlative CT images exhibit lytic-destructive lesions with bone expansion in the right radius, indicating a probable brown tumor (**D**). Visualization of the kidneys in the bone scan may imply a rapidly progressive nature of the disease and good functioning kidneys in a previously healthy middle-aged woman.

Hyperparathyroidism, especially the secondary type, is the most common cause of metabolic superscan (Figure 7) [81]. This superscan pattern is also seen in up to 17% of patients under chronic peritoneal dialysis [142], and its incidence is correlated with the duration of peritoneal dialysis, high levels of parathyroid hormone and alkaline phosphatase, and severity of osteoporosis. These cases are characterized by more pronounced mandible uptake and focal uptakes in ectopic calcifications and brown tumors and diffuse lung uptake secondary to hypercalcemia [143] (Figure 8). The superscan view can even manifest in the blood pool phase attributed to the “bone hunger” mechanism. The appearance of bone in 3-8 min is predictive of the superscan pattern in delayed imaging [144]. Superscan appearance rules out adynamic bone disease [145]. The administration of cinacalcet in patients with renal osteodystrophy can increase ^99m^Tc-MDP uptake in the lower extremities; otherwise, ^99m^Tc-MDP absorption in the heart and soft tissues can be due to coronary artery disease and hyperphosphatemia, respectively [38,146]. In cases where a patient with renal osteodystrophy suffers from systemic nephrogenic fibrosis, the superscan pattern is altered to diffuse muscle uptake, sometimes called “reverse superscan” [147]. In addition, renal dialysis-associated osteomalacia is caused by aluminum toxicity. When aluminum toxicity occurs, bone scans exhibit poor uptake and increased background activity, resulting from the inhibition of tracer uptake by bone. In other words, the aluminum inhibits osteoblasts to form any new osteoids. This effect persists as long as aluminum is present. However, following the removal of aluminum using deferoxamine, the superscan view becomes dominant [4].

Hyperthyroidism is another endocrine disorder that can cause metabolic superscan. In these patients, the remodeling cycle is shortened, leading to an increase in alkaline phosphatase and periosteal reaction. Thyroid acropachy may also appear with the clubbing and swelling of fingers and toes. Other signs of autoimmune hyperthyroidism, such as ophthalmopathy and dermopathy, may exist in these patients, and bone pain is usually improved by antithyroid drugs [75].

Acromegaly, associated with McCune–Albright syndrome in up to 20–30% of cases, can also cause metabolic superscan. In addition to metabolic superscan characteristics, prognathism and degenerative changes may be seen in the bone scan [148].

Osteomalacia, a condition of decreased bone mineralization, is another common cause of metabolic superscan [149]. It can be due to vitamin D deficiency, rickets, hypophosphatemia resistant to vitamin D, Crohn’s disease, Celiac disease [82], renal tubular acidosis, tumor-induced osteomalacia [89], gastrectomy, or hereditary reasons. Osteomalacia is also common in patients with chronic kidney disease due to aluminum intoxication. Despite reduced bone mineralization in patients with osteomalacia, osteoid formation, calcium deposition, bone turnover, and PTH level increase and cause superscan patterns. Additionally, the foci of increased uptake in pseudofractures (also known as Looser’s zones) may be seen in patients’ femoral neck and scapula, often resolving with the initiation of treatment [33].

Renal tubular acidosis (RTA) is one of the underlying conditions that can cause osteomalacia, probably due to impaired secretion of hydrogen ions into the renal tubules (distal RTA). Secondary to impaired hydrogen ion secretion due to the neutral electrical charge of urine, other electrolytes, such as calcium, are excreted, leading to increased bone turnover and osteomalacia [85]. Distal RTA can also be associated with primary Sjogren’s syndrome [150]. Fanconi syndrome, which is a reabsorption disorder of solutes, glucose, and amino acids in proximal tubules, can also result in osteomalacia [151].

Fibrogenesis imperfecta ossium (FIO) is another rare disease with osteomalacia appearance due to impaired mineralization. In these patients with a superscan pattern, the skull uptake is low, which is also called the beheaded appearance [93].

A superscan pattern can be also observed in other diseases and conditions, including exogenous consumption of recombinant PTH, hypervitaminosis D, vitamin C deficiency, fluorosis, primary hyperoxaluria, hematologic diseases, and rheumatologic/orthopedic diseases. In normocalcemic patients, chronic administration of recombinant PTH can cause a superscan pattern [83], as can hypervitaminosis D [76]. Vitamin C deficiency can also lead to this pattern [4]. Fluorosis is usually endemic in places with high levels of fluoride in the drinking water but can also result from chronic consumption of large amounts of brewed tea, inhalant abuse, eating toothpaste, or ingesting soil containing high amounts of fluorine [152]. This disease is presented with polyarthralgia, bone pain and deformity, vertebral ankylosis, and entrapment neuropathy. Radiologic findings vary from osteosclerosis, a coarse trabecular pattern, osteopenia of long bones, or increasing cortical bone thickness. Various mechanisms are involved in the superscan patterns in these patients, such as increased alkaline phosphatase and parathyroid hormone levels, as well as osteoblastic activity. Fluoride deposition in active ossification sites leads to the resistance of osteoid tissue to resorption by osteoclasts [72].

Primary hyperoxaluria, a rare autosomal recessive disease with three types, can lead to the conversion of glyoxylate to oxalate and deposition in the kidney, liver, and bone marrow, resulting in kidney and liver failure. The deposition of calcium oxalate crystals in the bone marrow around the joints of long bones leads to an inflammatory reaction that creates the appearance of polyarthritis. This deposit leads to an abnormal increase in bone mineral density. Renal failure in these patients can result in renal osteodystrophy, although significant uptake in the calvarium, mandible, and ribs is rare [80].

Hematologic diseases such as leukemia [32], lymphoma [11], systemic mastocytosis [90], myelofibrosis [25], aplastic anemia [74], Castleman disease [92], Waldenstrom macroglobulinemia [74], and multiple myeloma [18] can also cause a superscan pattern. In mastocytosis, new bone formation begins in the appendicular areas due to tumoral bone marrow infiltration, resulting in the thickening of long bone trabeculae and diffuse osteosclerosis [153]. This condition increases bone mineral density, bone turnover markers, and tryptase levels [154].

In leukemia and myelofibrosis (previously known as agnogenic myeloid metaplasia), increasing blood supply to the marrow or increasing bone surface due to peripheral bone marrow expansion can cause a superscan pattern [32]. Conditions such as renal osteodystrophy, vitamin D deficiency, hyperparathyroidism, breast cancer, lung cancer, prostate cancer, lymphoma, gastric cancer, leukemia, tuberculosis, and polycythemia vera have been suggested to be associated with myelofibrosis [155]. In addition to the superscan pattern, myelofibrosis can also present with other distinct patterns on a bone scan, including increased long bone uptake, especially around the knees, and multiple focal uptakes, which mimic diffuse metastases [155]. In severe cases, bone marrow imaging shows severely decreased uptake in the central marrow, especially the vertebrae [156]. Moreover, myelosclerosis, also known as a sclerosing extramedullary hematopoietic tumor, is a clinical entity close to myelofibrosis reported to cause superscan [88]. Castleman disease is also believed to increase bone turnover due to the release of cytokines and interleukin-6 [92]. Recently, two cases of Rosai–Dorfman disease with a superscan pattern have been described [95].

Various rheumatologic/orthopedic diseases such as osteopetrosis, polyostotic fibrous dysplasia [74], Paget’s disease [79], chronic familial hyperphosphatemia (juvenile Paget’s disease) [4], ankylosing spondylitis, and rheumatoid arthritis [4] can also cause a superscan pattern. Osteopetrosis leads to an increase in bone cortical thickness [71]. Polyostotic fibrous dysplasia can be part of McCune–Albright syndrome [157], and while it leads to an increased bone-to-soft-tissue uptake ratio, multiple foci of increased uptake and a heterogeneous uptake pattern are present and it is unlikely that a typical superscan pattern will be seen in these patients [158]. Paget’s disease, in polyostotic and advanced cases, may lead to a superscan pattern [4]. Chronic familial hyperphosphatemia is a rare genetic disease due to the osteoprotegerin mutation, causing fever, bone pain, skull expansion, bending of long bones, and pathologic fractures. Alkaline phosphatase levels are elevated, and the radiographic pattern is similar to Paget’s disease, with a symmetrical and generalized appearance occurring at a young age [4].

### 7.3. Distinguishing Metabolic Superscan from Metastatic Superscan

Differentiating metabolic and metastatic superscan patterns may pose a diagnostic challenge and sometimes may remain a diagnostic dilemma [159]. However, there are various characteristics that help in distinguishing between the two patterns [66] (Figure 6). One of the most noticeable differences is the symmetric bone uptake seen in metabolic superscan, whereas metastatic uptakes are usually patchy and asymmetric. Prominent calvarium, tie sternum, costochondral junction beading, and prominent mandibular uptake are evident in metabolic superscan [86]. In some cases, only a horizontal line appears instead of diffuse uptake in the sternum, known as the striped tie sign. Additionally, distal appendicular and periarticular uptake are more common in metabolic superscan [160].

Osteoblastic lesions are more common in metastatic superscan, while lytic lesions are more commonly seen in metabolic superscan. Patients with metastatic superscan typically have higher lactate dehydrogenase levels and may be asymptomatic or experience back pain, whereas high serum calcium levels and rib pain are usually seen in patients with metabolic superscan [13,66].

**Figure 9:**
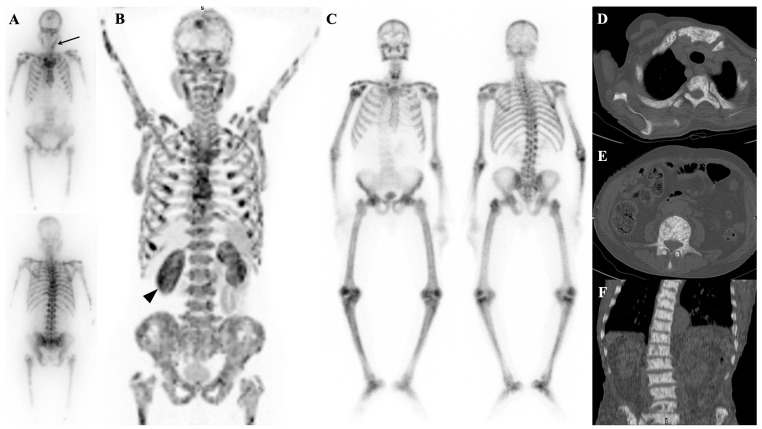
Double superscan, also known as super-superscan. A case of a prostate cancer patient with coexistent metastatic superscan and secondary hyperparathyroidism (PTH = 294 pg/mL, calcium = 7.6 mg/dL, Phosphorus = 2.6 mg/dL, and Creatinine = 1 mg/dL). Note the mandibular uptake (arrow) and absence of kidneys in the first post-treatment scan of ^177^Lu-PSMA-617 (**A**). Also, note the visualization of the kidneys in the pre-treatment ^68^Ga-PSMA-11 scan ((**B**), arrowhead). The underlying mechanism for discordant kidney uptake in the ^177^Lu-PSMA-617 and ^68^Ga-PSMA-11 is unknown. Another case of an elderly patient with a history of prostate cancer presents with progressive anemia and generalized weakness. A recent bone scan reveals atypical findings compared to the typical metastatic superscan observed in prostate cancer. Notable features include a prominent mandible and significant appendicular uptake (**C**). Upon examining fused images, widespread bone sclerosis is evident (**D**–**F**). The differential diagnoses considered are myelofibrosis/myelosclerosis or hyperparathyroidism superimposed on a metastatic superscan.

It is important to note that many findings of metabolic superscan, except for the non-visualization of kidneys, can also be seen in young individuals with a metabolically active skeleton [161]. In some cases, patients may exhibit both metabolic and metastatic superscan patterns, as seen in patients with extensive metastatic prostate cancer and chronic obstructive uropathy [96] or in those with prostate cancer and Paget’s disease [97] (Figure 9). There is also a rare possibility of the presence of secondary myelofibrosis secondary to prostate cancer [162] or thyroid cancer and parathyroid adenoma in patients with superscan [163].

Overall, a careful evaluation of clinical history, laboratory values, and imaging characteristics can aid in differentiating between metabolic and metastatic superscan patterns.

## 8. Conclusions

Superscan is a pattern that can appear on bone scintigraphy in both malignant and nonmalignant conditions. Although metastatic prostate cancer is the most common cause, other malignancies and nonmalignant conditions can also lead to this pattern. It is important for interpreters to consider a patient’s history and supplementary diagnostics to avoid false-negative results. Additionally, superscan should be identified promptly to allow for accurate diagnosis and appropriate management. In metabolic bone disease, the underlying cause can often be treated once diagnosed. Nuclear medicine physicians should be aware of superscan’s pathological causes, benign imitators, and clinical implications to avoid diagnostic pitfalls and prevent false-negative reports.

## Figures and Tables

**Table 1:** Proposed mechanisms for the appearance of the superscan pattern on bone scintigraphy.

Mechanism	Examples
**Chemisorption**	
Contributive factors include the following: - Increased regional blood flow - Osteoid formation - High bone turnover - Increased mineralizing bone surfaces	Myelofibrosis Osteomalacia Hyperthyroidism Hyperparathyroidism, Paget’s disease
**Bone marrow involvement**	
Increased tracer uptake due to disseminated bone marrow replacement by tumoral cells	Metastatic superscans
**Diminished urinary phosphate excretion**	
Secondary to increased bone resorption or some degrees of renal insufficiencies Increased phosphate levels probably resulting in elevated levels of hydroxyapatite crystals	Metabolic superscans (particularly hyperparathyroidism)
**Osteomalacia**	
Immature collagen fibers coupled to bone-seeking tracers	Hereditary metabolic superscans *

* e.g., McCune–Albright syndrome, Fanconi syndrome, osteopetrosis, and osteogenesis imperfecta.

**Table 2:** Etiologies of the superscan pattern on bone scintigraphy (references are given in the [brackets]).

Metastatic Superscan		Hematologic Disorders	Metabolic Superscan				
***More Common***	*Less* *Common*		*Endocrinopathies*	*Osteomalacia*	*Orthopedic Disorders*	*Rheumatologic Diseases*	*Miscellaneous*
Prostate cancer [15]	Urothelial carcinoma [10,68]	Multiple myeloma [18]	Hyperparathyroidism ** [69]	Vitamin D deficiency [70]	Osteopetrosis [71]	Rheumatoid arthritis [4]	Skeletal fluorosis [72]
Breast cancer [73]	Esophageal cancer [1,66]	Waldenstrom macroglobulinemia * [74]	Hyperthyroidism [75]	Hypophosphatemic rickets * [70]	Polyostotic fibrous dysplasia [74]	Ankylosing spondylitis [4]	Hypervitaminosis D [76]
Lung cancer [1]	Gastric cancer [77]	Leukemia [32]	Acromegaly [78]	Crohn’s disease	Paget’s disease [79]		Oxalosis [80]
	Colorectal cancer [66,81]	Lymphoma [11]		Celiac disease [82]	Juvenile Paget’s disease * [4]		Teriparatide injection [83]
	Nasopharyngeal cancer [84]	Myelofibrosis [25]		Renal tubular acidosis [85]			Aluminum toxicity [4,86]
	Neuroblastoma [87]	Myelosclerosis [88]		Tumor-induced osteomalacia [89]			Vitamin C deficiency [4]
	Ewing sarcoma [1]	Systemic mastocytosis [90]		Post-gastrectomy osteomalacia [91]			
	Medullary thyroid carcinoma [1]	Castleman disease [92]		Hereditary *** [93]			
	Melanoma [94]	Aplastic anemia [74]					
	Salivary gland tumor [1]	Rosai–Dorfman disease [95]					
	Pheochromocytoma [50]						
	Glioma [96,97]						
	Medulloblastoma [98]						

* Waldenstrom macroglobulinemia, hypophosphatemic rickets, juvenile Paget’s disease, and oxalosis are also known as lymphoplasmacytic lymphoma, vitamin D-resistant rickets, chronic congenital idiopathic hyperphosphatasemia, and primary hyperoxaluria, respectively. ** All forms (i.e., primary, secondary, or tertiary) have been associated with superscan. *** For example, fibrogenesis imperfecta ossium.

## Data Availability

Not applicable.
